# Editorial: Recent trends in infection biology and control of protozoan parasites

**DOI:** 10.3389/fcimb.2023.1292591

**Published:** 2023-09-20

**Authors:** Ragab M. Fereig, Charoonluk Jirapattharasate, Caroline F. Frey

**Affiliations:** ^1^ Division of Internal Medicine, Department of Animal Medicine, Faculty of Veterinary Medicine, South Valley University, Qena, Egypt; ^2^ Department of Pre-Clinic and Animal Science, Faculty of Veterinary Science, Mahidol University, Nakhon Pathom, Thailand; ^3^ Institute of Parasitology, Department of Infectious Diseases and Pathobiology, Vetsuisse-Faculty, University of Bern, Bern, Switzerland

**Keywords:** pathogenesis, diagnosis, vaccine, protozoa, drug, infection

Protozoan diseases including malaria and trypanosomiasis are among the most important parasitic causes of morbidity and mortality, both in immunocompetent and immunocompromised people. Cryptosporidiosis, toxoplasmosis, leishmaniosis, and giardiasis are zoonotic protozoan diseases that have been linked to severe outcomes, especially in immunocompromised individuals. Understanding the infection biology in naturally or experimentally infected animals is of paramount importance to control and prevent zoonotic transmission to humans. Protozoan infections also pose serious risks to personal, governmental, and international economy. Besides the direct and indirect costs of human infections, numerous protozoan infections in animals, such as coccidiosis and cryptosporidiosis in calves, toxoplasmosis in sheep and pigs, and neosporosis in pregnant heifers, can result in severe economic losses.

Other protozoan infections with medical and veterinary significance include those transmitted by ticks, such as babesiosis and theileriosis. The burden of protozoan diseases is expected to increase globally due to the lack of approved vaccines, ineffective treatments, the evolution of medication resistance, globalization and climate change, and a general neglect of parasitic diseases. Researchers must therefore make further efforts and devise novel strategies to control protozoan parasites and to address the current challenging scenario (Seed, 1996; Yaeger, 1996; Abdelbaky et al., 2021).

There is an urgent need for creative solutions to reduce the severe health risks and financial losses caused by protozoan diseases. Novel approaches to important problems in this sector, such as methods for developing effective vaccinations, accurate diagnostic tools, and drug discovery, have been targeted. Also, recent experimental approaches, novel solid data, and helpful information were sought to guide and assist the relevant research community.

In this Research Topic, four manuscripts have been accepted for publication based on relevance of the scope, novelty, significance, and high quality preparation by the authors. Three studies have investigated novel aspects of the vaccination and treatment regimen of *T. gondii* and malaria using murine models **(** Ezzatkhah et al., Shi et al., Zafar et al. **)**. Another study evaluated a useful tool to assess the interactions of protozoan parasites with their host cells **(** Koutsogiannis et al.). The paramount findings, points of strength, and the limitations of the studies will be discussed in brief in the following sections.

Ezzatkhah et al. investigated the inhibitory effect of *Curcuma longa* essential oil (CLE) on *T. gondii* RH tachyzoites *in vitro* using J774-A1 cells and *in vivo* using a BALB/c mouse model. CLE is a well-known spice usually used in foods and traditional medicine. A remarkable protective effect for CLE was noted both *in vitro* and *in vivo* against tachyzoites of *T. gondii* when atovaquone and saline were used as positive and negative controls, respectively. Also, CLE markedly decreased the oxidative stress markers, reduced the antioxidant enzymes and proinflammatory cytokines levels in the infected mice. No apparent cytotoxicity for vital organs was induced using CLE. This study demonstrated the usefulness of CLE in inhibition of *T. gondii* RH tachyzoites using several and diverse experiments. Mechanism of action and expected side effects were also assessed. However, this study did not examine the effect of CLE on the bradyzoites neither *in vitro* nor *in vivo*. Thus, this drug could alleviate clinical toxoplasmosis during parasitemia, but not in the latent form.

Treatment of microbial infections and other health problems with certain plants is widely used in traditional medicine. However, the use of some active principles derived from herbal components is a novel and promising research trend. Numerous herbal compounds have proven their efficacy against protozoan diseases and others. Nevertheless, these compounds are greatly influenced by several factors, e.g., climatic conditions, harvest season, and the part used, which may affect the biological features of herbs (Saedi Dezaki et al., 2016).

Differently from the study of Ezzatkhah et al. that used CLE as an herbal non-specific immune modulator against acute toxoplasmosis, Shi et al. assessed immunoprotective potentials of specific antigens derived from *T. gondii* itself. They developed cocktail DNA vaccines encompassing various dense granule proteins (GRA35, GRA42, GRA43). These antigens are critical for *T. gondii* for establishing chronic infection via formation of parasitophorous vacuoles. The effect of the developed vaccines was evaluated in a Kunming mouse model and demonstrated a protective potential against the highly virulent *T. gondii* RH strain (Type I) and the brain cyst-forming PRU strain (Type II). These findings were supported by the observation of specific IgG1 (marker for humoral immunity or T-helper 2 immunity) and IgG2 (marker for cellular immunity or T-helper 1 immunity). Also, the production of memory cells was confirmed via the increase of Gamma-Interferon (INF-γ), and activation of CD4+ and CD8+ cells. However, fostering the immune response is required to improve the protective potential of the vaccine candidate used. This can potentially be achieved via co-administration of a genetic adjuvant, for instance IL-33, IL-21/IL-15 and IL-7/IL-15.

In another study, Zafar et al. assessed the effect of cross-protection as a strategy against malaria disease using a co-infection mouse model. The authors infected the mice with *Babesia microti* (non-lethal strain) as a primary infection followed by *Plasmodium berghei* ANKA (lethal strain) as a challenge infection. Primary infection with *B. microti* alleviated the subsequent infection with lethal *P. berghei* when compared to a control group mono-infected with lethal *P. berghei.* Parasite burden in the organs, histopathological score, and serum cytokines of co-infected mice were significantly decreased, while the immune cell population was increased, compared to mice infected with *P. berghei* only. These findings were associated with an extended rate of survival in the co-infected group. This strategy might be beneficial in vaccine development because it is simple, easy, and cost effective compared to other strategies of vaccine development such as using recombinant DNA/proteins, or genetically edited parasites. Meanwhile, type of strain and infection dose and route of infection should be determined carefully to avoid aggravating the subsequent infection, and ethical concerns should be addressed.

Koutsogiannis et al. conducted a study published in this Research Topic, wherein they assessed a unique approach for examining host-*Toxoplasma* interactions. The present work involved an assessment of the lytic cycle of *T. gondii* through the utilization of distinctive refractive index (RI) and optical diffraction tomography (ODH) approaches employing 3D label-free optical diffraction holotomography (3D-ODH). The results of the study revealed that the application of this state-of-the-art imaging technology, known for its extensive content and exceptional resolution, exhibited its capacity to provide substantial real-time data. Additionally, this methodology has the benefits of being a non-invasive and non-toxic substitute for fluorescence microscopy. The researchers employed a technique known as three-dimensional optical deconvolution microscopy (3D-ODH) to evaluate and analyze host cells that were infected with both wild-type *Toxoplasma* strains and genetically modified CRISPR-Cas9 *Toxoplasma* mutant strains. The researchers highlighted the efficacy of 3D-ODH as a tool for evaluating the observed cellular modifications in volume, dry mass, or surface area in infected cells containing either parental or mutant parasites, in comparison to non-infected cells. The findings demonstrate the suitability of holotomography as a viable instrument for evaluating protozoan parasites and their interactions with host cells. In the present context, the limited availability of information calls for further research to explore the benefits and drawbacks of employing 3D-ODH technology in the field of protozoan parasites, with a specific focus on *T. gondii* infection.

Eventually, understanding the host-parasite interactions is the key for development of preventive or control measures against protozoan parasites. This Research Topic provides helpful guide and insightful information on recent approaches in the research fields of pathobiology and control of *T. gondii* and *P. berghei*, two highly fatal protozoan parasites. These findings can be exploited in developing novel vaccines and efficient drugs for the control and prevention of toxoplasmosis and malaria.

## Author contributions

RF: Conceptualization, Investigation, Project administration, Supervision, Validation, Writing – original draft, Writing – review & editing. CJ: Conceptualization, Investigation, Resources, Validation, Writing – original draft. CF: Conceptualization, Investigation, Resources, Validation, Writing – review & editing.
